# Characterisation of cell lines derived from prostate cancer patients with localised disease

**DOI:** 10.1038/s41391-023-00679-x

**Published:** 2023-06-01

**Authors:** Leire Moya, Carina Walpole, Fiona Rae, Srilakshmi Srinivasan, Inge Seim, John Lai, David Nicol, Elizabeth D. Williams, Judith A. Clements, Jyotsna Batra

**Affiliations:** 1 School of Biomedical Sciences, Faculty of Health, Queensland University of Technology, Brisbane, Australia; 2 Translational Research Institute, Queensland University of Technology, Brisbane, Australia; 3 Cancer Immunotherapies Group, Mater Research, Translational Research Institute, Brisbane, Australia; 4 Integrative Biology Laboratory, College of Life Sciences, Nanjing Normal University, Nanjing, China; 5 School of Biology and Environmental Science, Queensland University of Technology, Brisbane, Australia; 6 Australian Genome Research Facility Ltd, Gehrmann Laboratories, the University of Queensland, Brisbane, Australia; 7 Urology Department, Princess Alexandra Hospital, Brisbane, Australia; 8 Urology Unit, The Royal Marsden, London, UK; 9 Department of Surgery, St Vincent’s Hospital, University of Melbourne, Melbourne, Australia; 10 Center for genomics and Personalised Health, Queensland University of Technology, Brisbane, Australia

**Keywords:** Cancer, Medical research

## Abstract

**Background:**

Prostate cancer is a broad-spectrum disease, spanning from indolent to a highly aggressive lethal malignancy. Prostate cancer cell lines are essential tools to understanding the basic features of this malignancy, as well as in identifying novel therapeutic strategies. However, most cell lines routinely used in prostate cancer research are derived from metastatic disease and may not fully elucidate the molecular events underlying the early stages of cancer development and progression. Thus, there is a need for new cell lines derived from localised disease to better span the disease spectrum.

**Methods:**

Prostatic tissue from the primary site, and adjacent non-cancerous tissue was obtained from four patients with localised disease undergoing radical prostatectomy. Epithelial cell outgrowths were immortalised with human papillomavirus type 16 (HPV16) E6 and E7 to establish monoclonal cell lines. Chromosomal ploidy was imaged and STR profiles were determined. Cell morphology, colony formation and cell proliferation characteristics were assessed. Androgen receptor (AR) expression and AR-responsiveness to androgen treatment were analysed by immunofluorescence and RT-qPCR, respectively. RNA-seq analysis was performed to identify prostate lineage markers and expression of prostate cancer tumorigenesis-related genes.

**Results:**

Two benign cell lines derived from non-cancer cells (AQ0420 and AQ0396) and two tumour tissue derived cancer cell lines (AQ0411 and AQ0415) were immortalised from four patients with localised prostatic adenocarcinoma. The cell lines presented an epithelial morphology and a slow to moderate proliferative rate. None of the cell lines formed anchorage independent colonies or displayed AR-responsiveness. Comparative RNA-seq expression analysis confirmed the prostatic lineage of the four cell lines, with a distinct gene expression profile from that of the metastatic prostate cancer cell lines, PC-3 and LNCaP.

**Conclusions:**

Comprehensive characterization of these cell lines may provide new in vitro tools that could bridge the current knowledge gap between benign, early-stage and metastatic disease.

## Introduction

Prostate cancer was diagnosed in over 1.4 million men in 2020, and approximately 375,000 associated deaths were recorded worldwide in the same year [1], making this disease the most frequent non-skin cancer and the fifth deadliest cancer in men. Patient-derived cancer models are valuable tools to elucidate the underlying mechanisms of prostate cancer aetiology and progression. Moreover, understanding the basic features of clinically less significant disease is essential to uncover molecular sequence of events that lead to cancer progression, and to improve prognostic tools and treatments for localised disease. However, there are limited in vitro cell lines currently available to study early-stage disease.

LNCaP, PC-3 and DU145 are the most commonly used in vitro cell lines for prostate cancer research [2–4] and are derived from metastatic deposits [5–7]. Whilst these are important models for the study of advanced disease, they are not applicable for localised prostate cancer research. In addition, RWPE-1 and BPH-1 cell lines are frequently used as non-cancerous controls [4], but they are derived from normal and benign hyperplastic prostates, respectively [8, 9] and would not reflect any changes, subtle or otherwise, impacted by the tumour to the local prostatic microenvironment. A comprehensive two-part review of prostate cancer cell lines, where more than 110 prostate cancer cell lines were catalogued [10, 11], showed that over 90 of the cell lines were derived from either LNCaP, PC-3, RWPE-1, BPH-1 or their respective xenografts [4, 10–12], with more recent reviews reporting similar observations [4, 12–14]. Despite these models being important to research late stages of the disease, adjacent non-malignant epithelial prostatic cells and tumour cells from patients with localised disease are less available [15–17].

In conjunction with industry collaborators, we developed four cell lines from prostates surgically removed as part of localised prostate cancer management to bridge this gap. Two epithelial prostate cell lines were derived from the primary tumour and two from adjacent benign tissue. In this study, we describe some of the main molecular and phenotypic characteristics of these immortalised cell lines.

## Materials, methods and subjects

### Subjects and immortalisation of epithelial prostatic cell lines

Prostate tissue derived from the primary tumour or adjacent benign tissue was obtained from men undergoing radical prostatectomy for the management of localised prostate cancer in 2007. Cell lines were developed in collaboration with the now ceased UK-based biotechnology company Onyvax Ltd. Briefly, fresh tissues were minced and seeded in proprietary Onyvax medium to support epithelial cell growth. Cell lines were developed from outgrowths of these prostate epithelial cells in Keratinocyte Serum-Free Growth Medium media (KSFM Kit, ThermoFisher Scientific), and transformed with replication-defective human papillomavirus type 16 (HPV16) E6 and E7. Briefly, at passage 3, the propagated primary cells were infected with the HPV16 E6/E7 virus in the presence of 8 µg/mL of polybrene, as reported previously [18]. For sub-culturing, Accutase solution (Sigma-Aldrich) was used to detach the cells and serial dilutions were used to establish monoclonal cell lines. To promote cell homogeny through passages, all the cell lines originated and were each developed from one single parental clone. The cells were subsequently transitioned to Roswell Park Memorial Institute (RPMI-1640) media + 10% fetal bovine serum (FBS) (ThermoFisher Scientific) for routine use and experimental analysis. The study protocols were approved by the ethics committees Queensland University of Technology 1400000859, St Vincent’s Health 011/02 and Epworth Study 22102. All patients completed a consent form and the procedures followed were in accordance with the ethical standards of the responsible committee for human experimentation. Four immortalised cell lines (AQ0396, AQ0420, AQ0411, and AQ0415) were cultured for further characterisation.

### Short Tandem Repeats profiling

A minimum of 50 ng of genomic DNA was isolated from each cell line for Short Tandem Repeat (STR) profiling. Briefly, the cells were harvested after reaching ~85% confluence. Genomic DNA was extracted from cell pellets using the DNeasy Blood & Tissue Kit (QIAGEN,) as per the manufacturer’s instructions. The samples were submitted to the Analytical Facility at the Queensland Institute of Medical Research (QIMR Berghofer Medical Research Institute, Brisbane, Australia) to establish the DNA profile. Nine STR loci (*D5S818, D13S317, D7S820, D16S539, vWA, TH01, TPOX, CSF1PO* and *D21S11*), and the amelogenin sex-determining marker (*AMEL*), were amplified using the Applied Biosystems 3130XL Genetic Analyser platform (ThermoFisher Scientific). The genotype results were analysed using the GeneMarker V2.2.0. software (State College, PA, USA).

### Chromosomal ploidy analysis by karyotype

For karyotype analysis, the four cell lines were grown in T25 flasks until 70% confluence was achieved. After three washes with phosphate buffered saline (PBS), 10 μL/mL colcemid was added (Sigma Aldrich). This compound binds to tubulin, preventing the formation of mitotic spindles and thus helps to increase the number of cells in the metaphase state. The flasks were then submitted to Sullivan Nicolaides Pathology (Brisbane, Australia) for assessment. After disrupting the nuclear membrane, DNA was chemically fixed, and the structure and quantity of the chromosomes were analysed under a microscope and imaged by the pathology provider.

### Novel cell lines culture and cell morphology imaging

Cell lines were cultured as noted above alongside a panel of two prostatic benign (RWPE-1 and HPr-1), one benign prostatic hyperplasia (BPH-1) and one prostate stromal (WPMY-1) as well as four prostate cancer cell lines (LNCaP, RWPE-2, PC-3 and DU145) in parallel as references. All commercial cell lines were obtained from the American Type Culture Collection (ATCC, https://www.atcc.org/). These cells were cultured as follows: RWPE-1, RWPE-2 and HPr-1 were cultured in KSFM containing Bovine Pituitary Extract (BPE, 50 µg/mL as per ATCC instructions) and epidermal growth factor (EGF, 5 ng/mL, KSFM Kit, ThermoFisher Scientific). The remaining cell lines were cultured in phenol red free RPMI-1640 media containing glutamine (Glu^+^) + 10% FBS and 1% penicillin/streptomycin (ThermoFisher Scientific). All cell lines were maintained in a tissue culture incubator at 37 °C and 5% CO_2_. The cells were passaged upon reaching 70–80% confluence with 0.25% trypsin/EDTA (Invitrogen, ThermoFisher Scientific). For morphology studies, the cells were seeded into 6-well plates and imaged using a Nikon Eclipse Ti microscope (Nikon, Coherent Scientific).

### Cell proliferation assay by total DNA quantification

Cell proliferation was measured using the CyQuant assay (Invitrogen, Thermo Fisher Scientific), which quantifies nuclear cell DNA content as a direct representation of the total cell number. For this, 2000 cells/well were cultured in 100 µL RPMI-1640 (Glu^+^) + 10% FBS in a 96-well clear bottom black plate (Costar, Sigma-Aldrich). One plate for each time point was seeded and incubated at 37 °C for 96 hours. Every 24 hours, one plate was removed from the incubator and treated with 132 µL/well of CyQuant dye, following the manufacturer’s instructions. Fluorescence (520 nm) was then measured after excitation (480 nm) using a microplate reader PolarSTAR Optima (BMG Labtech). Eight technical replicates were included for every cell line and the experiment was performed independently three times.

### Cell invasion assay by sphere formation

The anchorage independent growth assay was performed for all four cell lines (AQ0411, AQ0415, AQ0396, and AQ0420) as well as RWPE-2 and PC-3 as positive controls. Briefly, the bottom layer was prepared with 1% (w/v) Ultra-pure LMP agarose (Invitrogen, ThermoFisher Scientific) dissolved in PBS and cooled to 40 °C. The agarose solution was diluted with RPMI-1640 (Glu^+^) + 10% FBS for a final 0.5% agar concentration and a total of 2 mL per well was added to a 6-well plate (Costar, Sigma-Aldrich). The agar was left for 15 minutes at room temperature to solidify before adding the top layer consisting of 0.66% Ultra-pure LMP agarose dissolved in PBS. An equal amount of media containing a single-cell suspension was immediately and carefully dispensed on top of the agar layer. After one hour incubation, allowing the top layer to solidify at room temperature, the plates were incubated at 37 °C. The new prostate cell lines were seeded at 20,000 cells/well while the controls (RWPE-2 and PC-3) were seeded at a final concentration of 10,000 cells/well. Different cell densities were used as starting points to account for the different cell growth rates observed in the proliferation assays. After three weeks at 37 °C, the colonies were pre-washed with PBS and stained with 0.5 mL fresh 0.01% crystal violet (Sigma-Aldrich) for 15 min at room temperature. Colonies were imaged with a Nikon Eclipse Ti microscope (Nikon, Coherent Scientific). Three technical replicates included in two independent experiments.

### AR expression analysis by immunofluorescence assay

The four cell lines (AQ0411, AQ0415, AQ0396 and AQ0420) were assessed for AR expression using immunofluorescence. Each cell line was plated at 60% confluence onto 6-well plates in triplicate, followed by fixing with 4% paraformaldehyde (Sigma-Aldrich) for 10–15 minutes at room temperature. Cells were incubated for 10 minutes in 0.25% Triton X-100 (Sigma-Aldrich) followed by three washes with PBS (5 minutes each). The fixed cells were incubated for 30 min in 2% BSA in PBS-Tween20 (BSA/PBST) solution followed by an overnight incubation with the primary AR antibody (1:100 dilution in 1% BSA/PBST, Santa Cruz sc-815, Abcam) at 4 °C on an orbital shaker. After three five-minute washes with PBS, the cells were incubated with Alexa Fluor 488 labelled secondary antibody (Goat Anti-Rabbit IgG H&L, ab150077, Abcam) at 1:500 dilution in 1% BSA/PBST for one hour at room temperature on an orbital shaker. Two washes with PBS, followed by a final wash with water, were performed prior to nuclear staining with 4′,6-diamidino-2-phenylindole (DAPI) and mounting in Prolong Gold (both from Life Technologies, Thermo Fisher Scientific).

### AR activity assay

#### Androgen treatment and controls

To confirm the expression of an active form of AR, the four new cell lines were treated with vehicle (20% ethanol) or with androgen (dihydrotestosterone, DHT, Sigma-Aldrich) as described previously [19]. The AR-positive cell line LNCaP was treated in parallel as a positive control. Briefly, cells were cultured in phenol red-free RPMI-1640 (Glu^+^) + 5% Charcoal Stripped Serum (CSS, Thermo Fisher Scientific) for 48 hours prior to the assay. Cells were seeded at 900,000 cells/well in 6 well plates (2 mL/well) and grown for 48 hours at 37 °C. Cells were then washed with PBS and fresh media was added followed by the treatment: either vehicle (20% ethanol) or 10 nM DHT for an additional 24 hours. RNA was subsequently isolated using the RNeasy Mini Kit (QIAGEN) following the manufacturer’s instructions.

#### RNA extraction, cDNA synthesis and RT-qPCR

Total RNA was reverse transcribed into cDNA using the SensiFAST cDNA synthesis Kit (Bioline) as per manufacturer’s protocol. Quantitative reverse transcription PCR (qRT-PCR) was performed in a MicroAmp Optical 384-well plate (Applied Biosystems, Thermo Fisher Scientific) and the amplification was measured with SYBR Green (Applied Biosystems, ThermoFisher Scientific). The primers used were as follows, *AR*: forward 5’-AAAAGAGCCGCTGAAGGGAA-3’, reverse 5’-GAAGACGACAAGATGGACAATTT-3’; *KLK3 (Kallikrein-3)* positive control: forward 5’-AGTGCGAGAAGCATTCCCAAC-3’, reverse 5’-CCAGCAAGATCACGCTTTTGTT-3’; *RPL32* housekeeping gene: forward 5’-CCTTGTGAAGCCCAAGA-3’, reverse 5’-GACTGGTGCCGGATGAACTT-3’ (Sigma-Aldrich). The qRT-PCR analyses comprised of three biological replicates, unless stated otherwise, and three technical replicates per plate. The assay was performed on a ViiA 7 Real-Time PCR System (Applied Biosystems, Thermo Fisher Scientific).

### Overall and prostate cancer gene expression landscape by RNA-seq analysis

#### RNA sample preparation

A minimum of 10 µg of total RNA was extracted in duplicate from the newly established (AQ0411, AQ0415, AQ0396, and AQ0420) and commercial (RWPE-1, RWPE-2, BPH-1, HPr-1, LNCaP and PC-3) prostate cell lines using the RNEasy Plus Mini kit (QIAGEN) as above. Briefly, cells were grown in T75 flasks (NUNC, ThermoFisher Scientific) until they reached 80% confluency. The extracted RNA was quantified using the NanoDrop 1000 Spectrophotometer (Thermo Fisher Scientific) and RNA integrity was assessed by Bioanalyser RNA Analysis (Agilent Technologies). All samples had a RIN value of 10, indicating an optimal sample quality.

#### RNA library generation and data analysis

RNA-seq library preparation and transcriptome sequencing of the ten prostate cell lines was performed at the Garvan Institute of Medical Research (Darlinghurst, Australia). RNA sequencing libraries were prepared using the Illumina TruSeq RNA Sample prep Kit (Illumina, Scoresby, Australia). Library preparations were sequenced on an Illumina HiSeq 2000 sequencer (Illumina) and 125 bp/150 bp paired-end reads generated using. RNA sequencing reads were mapped against the human genome (human assembly GRCh37/hg19, UCSC Genome Browser [20]) using TopHat v1.3.2 [21, 22], with annotated genes to perform transcript-guided mapping (UCSC Custom Tracks available in Supplementary Table 1).

#### Heat map generation

Read counts were obtained from TopHat-generated BAM files using SAMtools v0.1.19 [23] and raw gene counts were computed from TopHat-generated BAM files using feature Counts v1.4.5-p1 [24], counting coding sequence features of the UCSC hg19 gene annotation file (gtf). FeatureCounts output files were analysed using R (v.3.2.2). Briefly, raw counts were normalised by Trimmed Mean of M-values (TMM) correction [25, 26]. Library size-normalised read counts per million (CPM) were employed to generate scaled heatmaps (unsupervised hierarchical clustering by Euclidean distance) and the final heatmap was generated in R using heatmap.3 (available at https://goo.gl/Yd9aTY). Samples with no counts were assigned a log2 CPM values of -5. Count data and associated scripts are available at https://github.com/sciseim/onyvax_MS.

#### Differential gene expression between the ten cell lines

Generalized fold change (GFOLD) v1.1.2 [27] was employed to quantify gene expression levels. Briefly, read counts were obtained from TopHat-generated BAM files (see above) and differentially expressed genes were assessed by comparing the gene expression of all cell lines to a reference cell line (RWPE-1) as outlined in the GFOLD manual. Associated scripts and GFOLD output data are available at https://github.com/sciseim/onyvax_MS.

## Results

### Novel epithelial prostatic cell lines derived from localised prostate cancer patients

Herein, we present four immortalised cell lines derived from the primary adenocarcinomas or adjacent benign tissue from four prostate cancer patients aged 59–69 years. All four patients presented essentially low to moderate disease (Gleason Scores 6 ≤ 8 (Table 1). Regarding the Gleason score 8, the pathologist reported this patient (A0396) presenting with extensive Gleason Grade 3 and a small area of Gleason Grade 5. The two cancerous cell lines (AQ0411 and AQ0415) originated within primary tumours, while the two benign cell lines (AQ0396 and AQ0420) were derived from benign, non-cancerous cells adjacent to the primary prostate tumour. Patient AQ0411 had localised disease, while patient AQ0415 presented with extracapsular extension and seminal vesicle invasion. Both benign cell lines were derived from radical prostatectomy specimens containing adenocarcinomas with no seminal vesicle invasion, although patient AQ0396 had extensive perineural invasion.

**Table 1:** Pathology characteristics from the four immortalised cell lines derived from localised adenocarcinomas.

Patient	Cell line	Tissue Classification	Gleason score tumour	Number clones	Growth media	Pathology Notes
Patient 1	AQ0411	Hyperplasia, Adenocarcinoma	3 + 3	10	RPMI (Glu^+^) + 10%FBS	Nodular hyperplasia and adenocarcinoma of the prostate with minimal carcinoma. No extracapsular extension and no extension of the tumour into seminal vesicles. Foci of PIN present. Right base site.
Patient 2	AQ0415	Prostatic Adenocarcinoma	4 + 3	2	RPMI (Glu^+^) + 10%FBS	Extensive adenocarcinoma of the prostate involving most of the left lobe. Multiple foci of extra capsular penetration and seminal vesicle invasion. No evidence of lymph node metastasis. Left middle site.
Patient 3	AQ0396	Adjacent benign	3 + 5	5	RPMI (Glu^+^) + 10%FBS	Adjacent to prostatic adenocarcinoma tissue. Extensive GG 3 in right prostate extending from apex to base. One small area of GG5. Multifocal high-grade PIN. Extensive perineural invasion. ECE present and no seminal vesicles invasion. Surgical margins are clear. Right site.
Patient 4	AQ0420	Adjacent benign	3 + 4	5	RPMI (Glu^+^) + 10%FBS	Adjacent to prostatic adenocarcinoma tissue. Focal ECE with no seminal vesicle involvement. Tumour abutting posterior plane of resection in midline of mid-zone and right apex.

Cell lines were established from outgrowths of prostate epithelial cells in KSFM and transformed with HPV16 E6 and E7. All were later transferred to RPMI (Glu^+^) + 10%FBS for longterm use. *RP* Radical prostatectomy, *PCa* Prostate cancer, *ECE* Extracapsular extension, *GG* Gleason grade, *GS* Gleason score, *PIN* Prostatic intraepithelial neoplasia, *FBS* Fetal bovine serum, *Glu*^*+*^ Media supplemented with glutamine.

**Fig. 1:**
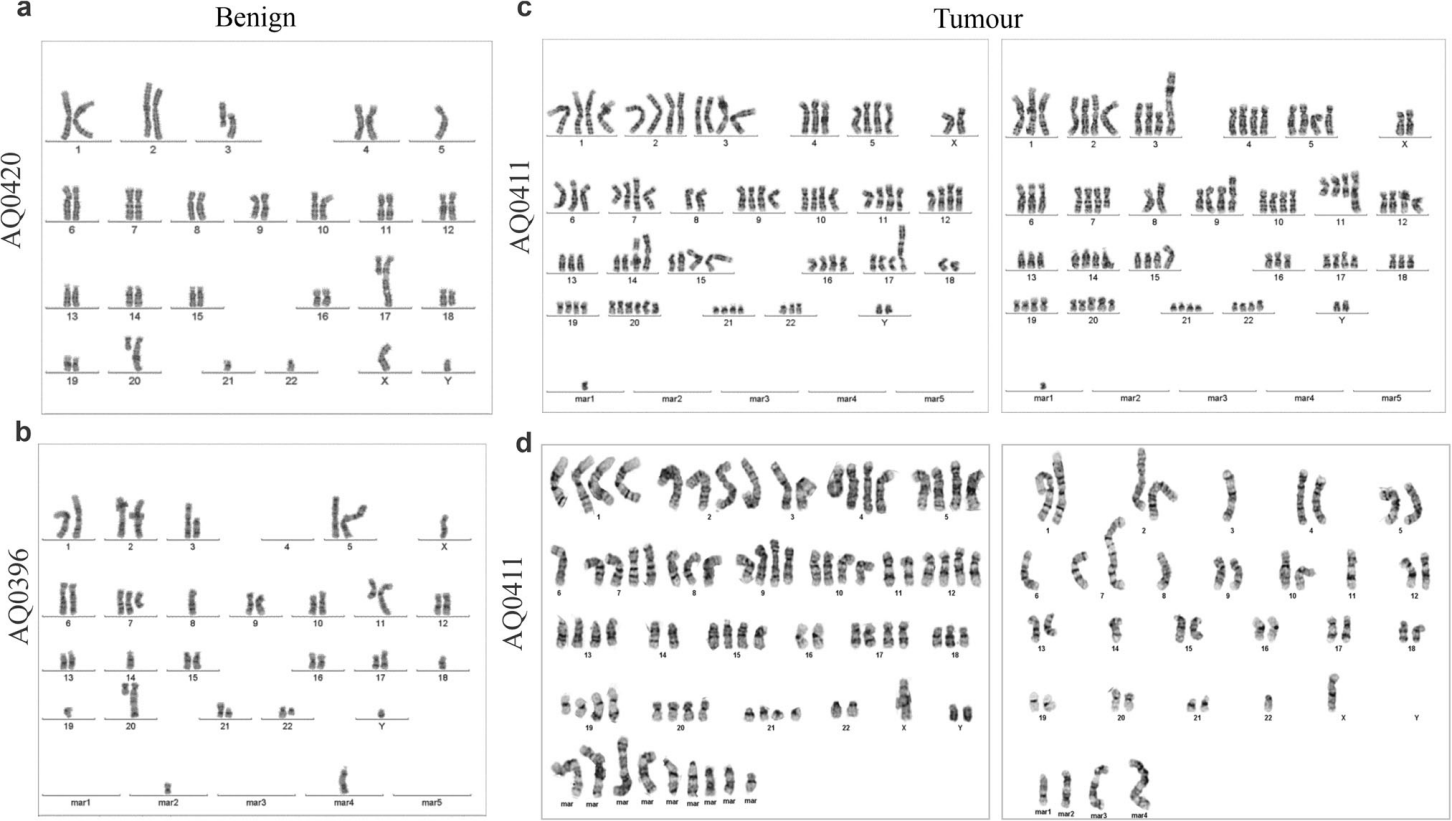
Karyotype of the primary benign and tumour-derived epithelial prostate cell lines. The analysis reveals a mostly diploid karyotype for the two benign cell lines (**a**, **b**) while tumour tissue derived cell lines (**c**, **d**) display abundant cell polyploidy. mar marker chromosomes not identifiable.

The human DNA profile of the ten STR markers analysed by us is presented for the four cell lines in Supplementary Table 2. This analysis aimed to identify their unique genomic signature so that during future in vitro work, they can be easily validated by their DNA fingerprint. Karyotyping was performed to assess the degree of chromosomal rearrangements and polysomy (Fig. 1). Analysis of the two cell lines derived from benign tissue show mostly diploid karyotypes, with some observable gains and losses (Fig. 1a, b). AQ0420 is mostly diploid except for chromosomes 5, 21, and 22, which are haploid. Translocation events were also present in chromosomes 3, 17, and 20. AQ0396 displays a diploid karyotype with some chromosomal restructure. For example, there is a loss of chromosome 4, while the cell line is haploid for chromosomes 8, 14, 18, and 19, with a trisomy of chromosome 7 and the gain of 2 marker (mar chromosomes - unidentifiable). Evidence of losses in chromosome 3 and gains in chromosomes 11 and 20 is also observed. By contrast, both cell lines derived from primary tumours show evidence of multiple polyploidy and major chromosomal disruption and gain of several unidentifiable marker chromosomes (Fig. 1c, d). All chromosomes for AQ0411 presented polyploidy, with various translocation and telomere fusion events. A mixture of cells is observed in AQ0415, with some presenting mainly polyploidy chromosomes and a large array of mar chromosomes and others showing mainly diploid and haploid chromosomes with few gains of mar chromosomes.

### Cell morphology, invasion and proliferation analysis

**Fig. 2:**
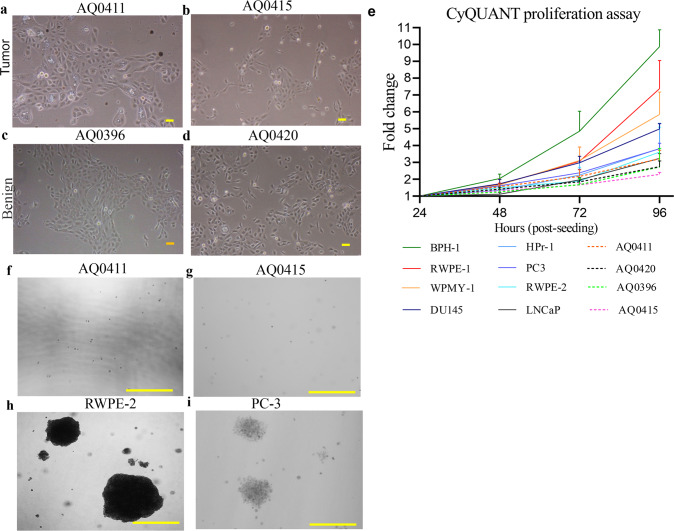
Morphological, proliferative, and invasive characterisation. **a**–**d** Phase contrast images of prostatic and localised prostate cancer derived cell lines, 10x magnification, scale bar 100 µm. All show epithelial morphology. **a**, **b** Tumour (AQ0411, AQ0415), **c**, **d** Benign (AQ0396, AQ0420) cell lines. **e** Cell proliferation assay of the new lines (dashed lines), benign (WPMY-1, RWPE-1, BPH-1, HPr-1) and cancerous (LNCaP, RWPE-2, PC-3 and DU145) prostate cell lines. Cell lines represented in the graph follow the order in the legend. Mean ± SEM, *n* = 3, statistical significance for all these assays were analysed by Friedman test with Dunn’s multiple comparison test. **f**–**i** Soft agar assay with crystal violet staining. **f**, **g** AQ0411 and AQ0415. **h**, **i** RWPE-2 and PC-3, 20x magnification, scale bar 500 µm. Three replicates were included unless otherwise indicated from two independent experiments.

Under phase-contrast microscopy, the cell lines show a typically epithelial-like cobblestone morphology (Fig. 2a–d). Total DNA quantification detection at 24 hour post-seeding intervals shows the different proliferative rates of the tested cell lines (Fig. 2e). They are considerably slower than BPH-1, RWPE-1, and WPMY-1, all of which exhibit the highest cell division rates in this study. HPr-1, PC-3, and RWPE-2 have an overlapping growth rate at 96 hours, while our new cell lines group together below and alongside LNCaP. The growth rate of the metastatic DU145 cell line is in between these two groups. To determine if the two cancerous cell lines derived from localised prostate cancer (AQ0411 and AQ0415) had developed adhesion-independent capabilities, colony formation assays were performed. Neither of these two cancerous cell lines formed colonies in suspension ( > 150 µm) (Fig. 2f, g). The two benign cell lines (AQ0396 and AQ0420) also showed no colonies (data not shown). Both controls (RWPE-2, and PC-3) grew in an anchorage-independent manner as reported previously [28, 29] (Fig. 2h, i respectively).

### Overall landscape and prostate cancer related gene expression

Comparative gene expression analysis of our four cell lines and six (BPH-1, HPr-1, LNCaP, RWPE-1, PC-3 and RWPE-2) commercially available prostate cell lines was performed using RNA-seq data. RNA-seq produced approximately 35–43 million reads, with 51–59% of reads mapping to an annotated gene (GRCh37/hg19, Supplementary Table 3).

**Fig. 3:**
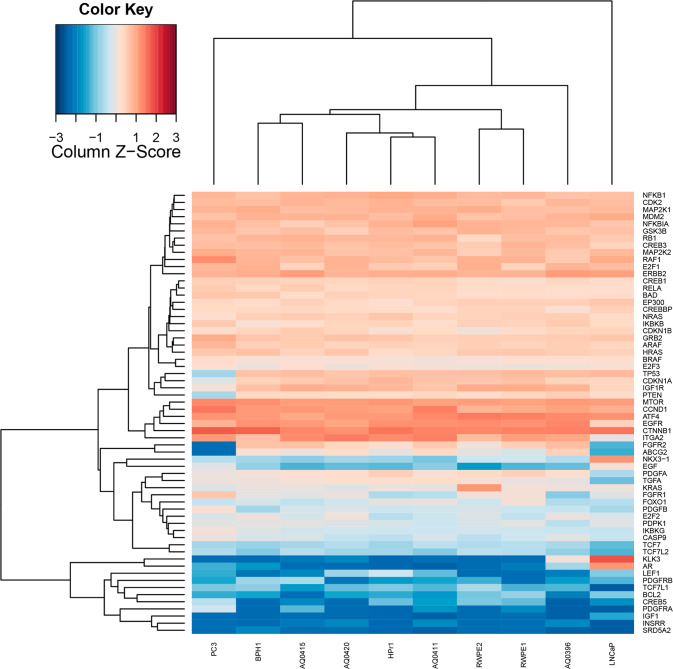
Expression characterisation with KEGG prostate cancer pathway (map05215). RNA-seq library size-normalised read counts per million (unsupervised hierarchical clustering by Euclidean distance). Scaled log_2_ transformed normalized counts (*Z*-score) are plotted in blue–red colour, with red indicating high expression and blue indicating low expression. Cell lines derived from localised prostate cancer patients from the primary site: AQ0411, and AQ0415 or benign tissue: AQ0396, AQ0420. These cluster away from the two metastatic derived LNCaP and PC-3 cell lines. KLK3 Kallikrein 3, PSA Prostate specific antigen. DHT Dihydrotestosterone. KEGG Kyoto Encyclopedia of Genes and Genomes.

We performed the same analysis in the context of genes associated with prostate cancer tumorigenesis by assessing the prostate cancer pathway gene expression in the Kyoto Encyclopedia of Genes and Genomes [30, 31] (map05215) (Fig. 3). The four new cell lines show a distinct gene expression profile from those derived from metastatic patients, LNCaP and PC-3. The expected high expression levels of *AR* and *KLK3* (303.0 and 1,541.5 counts per million, CPM, respectively) are observed in LNCaP (Fig. 3). Interestingly, a similar expression pattern is observed in the case of the [adjacent] benign cell line AQ0396 (3.0 and 18.8 CPM for *AR* and *KLK3*, respectively), while the other cell lines have close to zero counts.

**Fig. 4:**
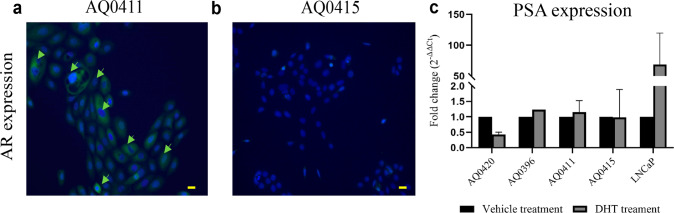
Androgen receptor and KLK3/PSA expression analysis. **a**, **b** Immunofluorescence AR assay with **a:** cancerous AQ0411 and AQ0415. Moderate AR expression was observed in AQ0411 (yellow arrows) in growth media (RPMI-1640 (Glu^+^) + 10% FBS). Green stain represented by AR antibody. Blue stain represented by nuclear blue (DAPI). Scale bar 100 µm. **c** PSA expression after androgen (10 nM DHT) treatment by qRT-PCR analysis. Positive control LNCaP cells showed a sharp increase of PSA expression as expected. Vehicle: 20% ethanol and treatment lasted for 24 h. Mean ± SEM, *n* = 3, except for AQ0396 *n* = 1 (Mann Whitney t-test).

A functional androgen receptor would be expected to drive *KLK3* (PSA) gene expression in prostate-derived cells. Due to the significant role that AR and androgens play in prostate cancer initiation and development, AR expression and androgen response assays were performed. IF analysis on the two cancerous cell lines shows that AQ0411 has a low AR expression while AQ0415 shows none (Fig. 4a, b, respectively). Additionally, to further determine AR activity, PSA expression levels were analysed after DHT treatment. The results suggest that none of the four cell lines expresses an active form of AR since *PSA*/*KLK3* levels did not change with treatment (Fig. 4c). The positive control LNCaP exhibited a sharp increase in *KLK3* expression after treatment, as expected [5]. The relative expression of *KLK3* observed in the four cell lines towards the housekeeping gene (*RPL32*) were within the following ranges: (∆C_t_)_vehicle_ = 15.7–17.30 and (∆C_t_)_DHT_ = 15.99–17.48, while values for the positive LNCaP control are (∆C_t_)_vehicle_ = 4.7–5.2 and (∆C_t_)_DHT_ = 10.7–8.

### Characterisation of prostatic and prostate cancer tissue lineage genetic markers

To confirm the presence of heterogeneous cell morphology typical of prostate cancer disease [32, 33], markers for stem, basal, intermediate and luminal-secreting cells were examined in the four cell lines alongside six established commercial cell lines as reference (Table 2; Supplementary Figure 1). Except for LNCaP and PC-3, all the cell lines express stem, basal, intermediate, luminal, and prostate cancer markers. LNCaP and PC-3 lack gene expression of some or all of the stem cell (*CD44*) and basal (CK5, *KRT5*, CK14, *KRT14*, p63, *TP63*) markers assessed. Expression of additional stem cell markers, a2-integrin (*CD49B*) and BCRP (*ABCG2*) was lower in the metastatic LNCaP, and PC-3 cells and higher expression observed for the four new cell lines (Supplementary Table 4). Prostate Specific Stem Antigen (*PSCA*) was positive in AQ0396, AQ0420, AQ0411, LNCaP and RWPE-2. *AR* and *KLK3/PSA* are highly expressed in LNCaP as expected. *AR* expression in AQ0396 was detected, but this was comparatively very low: an RPKM of 0.77 compared to 67.5 in LNCaP. Only LNCaP and the benign AQ0396 showed *KLK2* expression. Also of note, although only the benign cell line AQ0420 expresses *ERG*, several other key genes expressed in the prostate and during tumorigenesis – *AMACR*, *GSTP1*, *NOTCH1*, *PCNT1*, *PTEN* and *NES* – are expressed in all four newly derived cell lines.

**Table 2:** Expression of prostatic tissue lineage and prostate cancer markers by RNA-seq analysis.

		Non-cancerous				Cancerous					
		BPH-1	RWPE-1	AQ0396	AQ0420	AQ0411	AQ0415	HPr-1	LNCaP	RWPE-2	PC-3
Stem	*CD44*	+	+	+	+	+	+	+	**−**	+	+
	*CD133*	**−**	**−**	**−**	**−**	**−**	**−**	**−**	**−**	**−**	**−**
Basal	*CK5*	+	+	+	+	+	+	+	**−**	+	**−**
	*KRT14* (CK14)	+	−	+	+	+	+	+	**−**	+	**−**
	*TP63* (p63)	+	+	+	+	+	**−**	+	**−**	+	**−**
Intermediate	*KRT8* (CK8)	+	+	+	+	+	+	+	+	+	+
	*PSCA*	**−**	**−**	+	+	+	**−**	**−**	+	+	**−**
Luminal	*KRT18* (CK18)	+	+	+	+	+	+	+	+	+	+
	*AR*	**−**	**−**	−^*^	**−**	**−**	**−**	**−**	+	**−**	**−**
	*KLK3* (PSA)	**−**	**−**	+	**−**	**−**	**−**	**−**	+	**−**	**−**
Prostate tumorigenesis	*KLK2*	**−**	**−**	+	**−**	**−**	**−**	**−**	+	**−**	**−**
	*AMACR*	+	+	+	+	+	+	+	+	+	+
	*ERG*	**−**	**−**	**−**	+	**−**	**−**	**−**	**−**	**−**	**−**
	*GSTP1*	+	+	+	+	+	+	+	−	+	+
	*NOTCH1*	+	+	+	+	+	+	+	+	+	+
	*PCA3*	**−**	**−**	**−**	**−**	**−**	**−**	**−**	+	**−**	**−**
	*PCMT1*	+	+	+	+	+	+	+	+	+	+
	*PTEN*	+	+	+	+	+	+	+	+	+	+
	*NES*	+	+	+	+	+	+	+	**−**	**−**	+

^*^Very low levels (RPKM < 1), suggesting that the cell line is AR-negative. ‘+’ indicate positive expression, and ‘−’ indicate negative expression. *RPKM* Reads Per Kilobase of transcript per Million mapped reads.

Prostate cancer gene markers were analysed to confirm the heterogeneous cell morphology typical of prostate cancer.

## Discussion

Since their establishment many decades ago, the most commonly used cell lines for prostate cancer research are all derived from metastases: LNCaP from the lymph node [5], PC-3 from the vertebra [6] and DU145 from the brain [7]. Moreover, two non-cancerous prostatic cell lines frequently included in prostate cancer research as benign controls (RWPE-1 and BPH-1) were not derived from prostate cancer patients. Instead, they originated from a prostate removed during a cystoprostatectomy for bladder cancer management and a patient with benign prostatic hyperplasia, respectively [9, 34]. To our knowledge, immortalised prostate cancer cell lines originated from the prostatic tissue of prostate cancer patients with localised disease are not accessible to the research community [4, 10–12, 35, 36]. For instance, RC-77N/E and RC-77T/E were derived from an African American patient with primary disease (tumour and paired adjacent benign tissue, respectively) [37]. Despite these cell lines used in a later publication [38], we were not able to locate them available in an Authenticated Cell Repository. The malignant prostatic CA-HPV-10 originated from a localised prostate cancer patient, presented a Gleason score of 8 [39], while the two new prostate cancer cell lines, AQ0411 and AQ0415, scored 6 and 7, respectively. Similarly, IGR-CaP1 [40] and RC-165N/hTERT [41], cell lines derived from localised prostate cancer patients are also not openly available. Non-malignant prostatic cell lines such as PWR-1E [16] or EP156T [15] differ from the presented four cell lines because they originated from a benign hyperplastic prostate tissue or showed an abnormal karyotype in subsequent studies [17], respectively.

This study has characterised cell lines, derived from radical prostatectomy specimens obtained from four men with localised prostate adenocarcinoma, in monolayer cell cultures. All cell lines exhibit an epithelial cell morphology and proliferate at similar rates. Chromosomal ploidy imaging shows inter- and intra-tumour heterogeneity in both of the tumorigenic cell lines, typical of many cancers [42], including prostate cancer [32]. The karyotyping results show that the two cancerous-derived cell lines present more chromosomal rearrangements events and a number of unidentifiable marker chromosomes than the two benign-derived tissues, with cells presenting a mixture of chromosomal rearrangements and ploidies. This is expected since cancer cells exhibit chromosomal instability even when originating from single clones or immortalised with other methods than HPV [43–46]. Since all were immortalised under the same protocol, these differences suggest that the genomic abnormalities may result from the tumourigenic transformation during disease initiation and development in the patient rather than a product of the immortalisation process.

Neither of the two cancerous cell lines can form colonies in an anchorage-independent manner, despite this process being known to be a tumorigenesis cell hallmark [47]. We initially considered the possibility that these cells may still possess their anoikis programmed cell death signalling pathway. This signalling process is typical of non-malignant cells, activated when cells lack an adequate cell adhesion surface [48]. Instead, metastatic cells acquire anoikis resistance, allowing them to detach from the original tissue or organ and grow in a distant extracellular matrix environment, initiating metastasis [49]. To confirm this, we performed a Gene Enrichment analysis (gsea-msigdb.org/gsea/msigdb/collections.jsp) to determine the expression of 29 genes associated with anoikis in these cell lines (Supplementary Fig. 2). Similar gene expression patterns were observed amongst the four cell lines presented here and the other prostate-derived cell lines. High expression of *ITGB1, CAV1* and low expression of *CRYBA1, CEACAM5* were observed in all, suggesting the lack of anchorage-independent growth of the two cancer-derived cells is due to different mechanisms than lack of anoikis resistance. Moreover, proliferation may be influenced by other factors such as seeding density that determines the cell-to-cell contacts, affects proliferative ability of the cell lines and the selection of subclones during passaging.

A heterogeneous prostatic lineage, typical of the early stages of prostate cancer disease [50] is observed in the four novel cell lines. By contrast, LNCaP and PC-3 lack all basal markers and PC-3 has two stem lineage markers. Additionally, most of the novel cell lines (AQ0396, AQ0420 and AQ0411) show *PSCA* expression. This marker is highly expressed in prostatic tissue compared to other tissues [51, 52]. While the up-regulation of *PSCA* has been extensively studied in high-grade and later stages of the disease [53–56], what drives this initial upregulation in the primary tissue, and indeed in pre-cancerous stages, is less well understood. However, technological limitations of the platforms used to determine the gene signatures, such as the bias imposed due to the choice of distance and clustering methods to compare different groups [57], need to be considered that may have impacted some of our analyses above. Therefore, the cell lines presented herein could be a valuable tool to elucidate the role of *PSCA* in these early stages. On this note, a study comparing data sets from the tumour and adjacent non-cancerous and healthy tissues from eight cancers (including prostate cancer) found that shared signalling pathways across tissues were activated in the adjacent non-cancerous cells, proving molecular changes associated with a pre-cancerous stage [58].

None of the four cell lines is AR-responsive after androgen treatment and no expression was observed neither by immunofluorescence nor RNA-seq, with the exception of the cancerous AQ0411 and benign AQ0396 cells. AQ0411 cell line exhibited low AR expression only in our immunofluorescence assays, while AQ0396 showed low AR expression in the RNA-seq analysis. These discrepancies are likely due to the polyclonal origin of this cell line, or driven by HPV integration as a result of the HPV16 E6/E7 immortalization process, one of the standard method for immortalization, at the time when these new cell lines were established [18, 59]. The loss of AR expression in immortalised non-malignant prostatic epithelial cell lines grown in monolayers is an appreciated limitation of their use [60]. It has been shown that when cultured on Matrigel and stromal-enriched media, AR expression can be stimulated in primary epithelial cancer cells [61]. Despite these conditions being useful in some experimental settings, this would not be for high throughput screening assays since the media requires a multi-step process to culture the stromal cells, and the epithelial fraction requires growth in KSFM media. The lack of expression of an active AR in these cell lines could provide an opportunity to ectopically over-express the different *AR* reported isoforms, improving our understanding of their roles in tumour initiation and development. Furthermore, *AR* expression declines after cell passaging [62] and the most commonly used prostate cancer cell line for AR studies, LNCaP [2–4], expresses a mutated form of the gene (T877A) that can modify its ligand-binding site and signalling [63]. None of the remaining commonly used prostate cancer cell lines that express the wild-type AR isoform (RWPE-2, DUCaP, LAPC, PC346 and VCAP) originate from the primary site [2]. The commonly-used AR-positive benign cell lines, RWPE-1, PWR-1E and RC-165N/hTERT [2] originated from adjacent or contralateral benign tissue – in contrast to AQ0396 and AQ0420. This could be relevant, since phenotype analysis and transcriptome expression changes following experimental modulation of *AR* expression/isoforms may help elucidate the AR-dependent molecular mechanisms that contribute to the initiation and development of the disease. Interestingly, in a study where samples from primary prostate cancer and benign prostatic hyperplasia were compared, three *AR* isoforms were dysregulated, and their opposing expression profiles correlated with disease aggressiveness [64]. However, it is also possible that these cell lines can express *AR*, and other genes, when growing under different conditions than a monolayer. In this study, we have measured AR-response by expression analysis of *PSA/KLK3*. However, PSA expression is critically dependent on cell morphology. PSA expression is observed in tall columnar cells of the prostate gland and often high in cells which are larger and well differentiated [65]. We observed the translocation of AR signal in the AQ0411 cell line (Fig. 4A), indicating a functional AR in these cells. Testing AR-response by other alternative markers may be more appropriate for the four cell lines. Reports of gene expression profile changes have been described in other cancer cell lines when growing as monolayers, spheroids or xenograft models [66–68]. Monolayer cultures represent only tumour cells, being grown in the absence of a microenvironment, in contrast to 3D spheroids or xenograft models. Thus, they have different transcriptomes to those observed in vivo [69–71]. Using current approaches, 2D cell cultures are particularly suitable for high throughput assays such as for drug development, as this format is highly reproducible, allows, for long term cultures, and minimises cost [72, 73]. The development of new technologies and novel approaches is enabling the incorporation of more complex culture systems, some of which will be able to be translated to large, high content screening platforms.

To conclude, the cell lines characterised in this study derived from localised prostate adenocarcinoma (AQ0411 and AQ0415) and adjacent benign-tissue (AQ0396 and AQ0420), have the potential to assist to further broaden research on early-stage prostate cancer and its impact on the surrounding benign prostate. Indeed, our preliminary data suggests these cell lines have the potential to further delineate between indolent and what may evolve towards clinically significant disease. This will require further analysis to determine which clinical states are most closely represented by these cell lines and whether more complex culture conditions such as 3-dimensional approaches or co-culture with other relevant cell types will also be useful. Nevertheless, this study has several limitations, such as the effect of the cell passage number on the genetic and molecular characteristics of the cell lines, or lack of data showing their ability to form tumours in xenograft mice models. We intend to deposit these cell lines in a publicly accessible Authenticated Cell Repository so additional investigation of these cell lines in 3D spheroids and pre-clinical models can be assessed and their usefulness as models of early prostate cancer can be further elucidated.

## Supplementary information

Additional Supplementary Material

Supplementary Table 1

Supplementary Table 2

Supplementary Table 3

Supplementary Table 4

Supplementary Figure 1

Supplementary Figure 2

## Data Availability

The RNA-seq data from which gene expression has been analysed has been provided in Supplementary Table 1 and can be freely accessed after uploading the information in the UCSC Genome Browser (https://genome.ucsc.edu). Once published we intend to make the cell lines available through Authenticated Cell Repositories.
