# Supplementary material for: Characterisation of cell lines derived from prostate cancer patients with localised disease
Source: Prostate Cancer Prostatic Dis. 2023 Jun 1;26(3):614–24. doi: 10.1038/s41391-023-00679-x (PMC10449630; doi:10.1038/s41391-023-00679-x)
Supplement: Supplementary file 1 — Additional Supplementary Material [file 41391_2023_679_MOESM1_ESM.docx]

**Supplementary Table Legends**

**Supplementary Table 1: RNA-seq UCSC custom tracks.**

**Supplementary Table 2: DNA profiling of commercial and novel prostate cell lines.** The four prostate cell lines show a unique number of repeats in their short tandem repeat (STR) profiles.

**Supplementary Table 3:** **RNA-seq performance summary.** The four cell lines and six commercial prostate cell lines performed similar in our RNA-seq analysis, including number of reads and 51-59% of reads mapping to an annotated gene (GRCh37/hg19).

**Supplementary Table 4: Expression of *ITGA2* and *BCRP* genes by RNA-seq analysis.** Expression of the genes in counts per million (CPM).

**Supplementary Figure Legends**

**Supplementary Figure 1: Expression of prostatic tissue lineage and prostate cancer markers.** Heatmap showing differences in the expression of the genes involved in the prostate cancer hallmarks for the HPr1, RWPE1, AQ0411 RWPE2, AQ0420, BPH1, AQ0396, AQ0415, PC-3 and LNCaP cell lines.

**Supplementary Figure 2: Gene enrichment ontology analysis of 29 genes associated with anoikis regulation.** The results show the benign and localised disease derived cell lines have an overall similar expression pattern compared to the PC-3 and RWPE-2 cell lines, for the genes analysed, suggesting they have also acquired anoikis resistance (gsea-msigdb.org/gsea/msigdb/collections.jsp).
