# Supplementary material for: Characterisation of cell lines derived from prostate cancer patients with localised disease
Source: Prostate Cancer Prostatic Dis. 2023 Jun 1;26(3):614–24. doi: 10.1038/s41391-023-00679-x (PMC10449630; doi:10.1038/s41391-023-00679-x)
Supplement: Supplementary file 3 — Supplementary Table 2 [file 41391_2023_679_MOESM3_ESM.docx]

|  | **Tumour derived** | | **Non-tumour derived** | |
| --- | --- | --- | --- | --- |
| Markers | **AQ0411** | **AQ0415** | **AQ0396** | **AQ0420** |
| **TH01** | 9.3 | 6,9 | 6,9.3 | 7,9.3 |
| **D21S11** | 29,31 | 30,31 | 31 | 28,29 |
| **D5S818** | 10,13 | 11 | 11,12 | 11,12 |
| **D13S317** | 8,13 | 12,13 | 10,11 | 8,11 |
| **D7S820** | 8,10 | 8,9 | 10,12 | 8,9 |
| **D16S539** | 13,14 | 11,13 | 12 | 12 |
| **CSF1PO** | 11,12 | 12,11 | 10,12 | 10,13 |
| **AMEL** | X,Y | X,Y | X,Y | X,Y |
| **vWA** | 17 | 14,17 | 14,18 | 14,19 |
| **TPOX** | 8 | 8 | 8 | 9,11 |
