# Supplementary material for: Characterisation of cell lines derived from prostate cancer patients with localised disease
Source: Prostate Cancer Prostatic Dis. 2023 Jun 1;26(3):614–24. doi: 10.1038/s41391-023-00679-x (PMC10449630; doi:10.1038/s41391-023-00679-x)
Supplement: Supplementary file 4 — Supplementary Table 3 [file 41391_2023_679_MOESM4_ESM.docx]

|  |  | **Reads assigned to annotated genes (hg19)** | |
| --- | --- | --- | --- |
| Cell line | Number of reads | Number | % |
| AQ0396 | 35,505,671 | 21,076,617 | 59% |
| AQ0411 | 35,146,142 | 19,771,106 | 56% |
| AQ0415 | 41,494,960 | 22,129,035 | 53% |
| AQ0420 | 38,335,081 | 22,628,955 | 59% |
| BPH-1 | 39,528,337 | 22,959,396 | 58% |
| HPr-1 | 43,949,484 | 25,956,285 | 59% |
| LNCaP | 36,418,332 | 18,448,242 | 51% |
| PC-3 | 37,321,210 | 21,121,873 | 57% |
| RWPE-1 | 39,390,558 | 20,514,274 | 52% |
| RWPE-2 | 38,23,7071 | 20,897,335 | 55% |
