# Supplementary material for: Characterisation of cell lines derived from prostate cancer patients with localised disease
Source: Prostate Cancer Prostatic Dis. 2023 Jun 1;26(3):614–24. doi: 10.1038/s41391-023-00679-x (PMC10449630; doi:10.1038/s41391-023-00679-x)
Supplement: Supplementary file 5 — Supplementary Table 4 [file 41391_2023_679_MOESM5_ESM.docx]

| Cell line | *CD49B (ITGA2)* | *ABCG2 (BCRP)* |
| --- | --- | --- |
| AQ0411 | 9.33 | 5.42 |
| AQ0415 | 9.00 | 4.06 |
| AQ0420 | 9.66 | 5.24 |
| AQ0396 | 7.64 | 5.83 |
| BPH1 | 6.51 | 4.56 |
| HPr1 | 8.66 | 3.70 |
| LNCaP | 3.80 | -0.92 |
| PC-3 | 7.67 | -5,00 |
| RWPE-1 | 8.17 | 3.06 |
| RWPE-2 | 6.73 | 3.69 |
