# Supplementary figures and images for: Characterisation of cell lines derived from prostate cancer patients with localised disease
Source: Prostate Cancer Prostatic Dis. 2023 Jun 1;26(3):614–24. doi: 10.1038/s41391-023-00679-x (PMC10449630; doi:10.1038/s41391-023-00679-x)

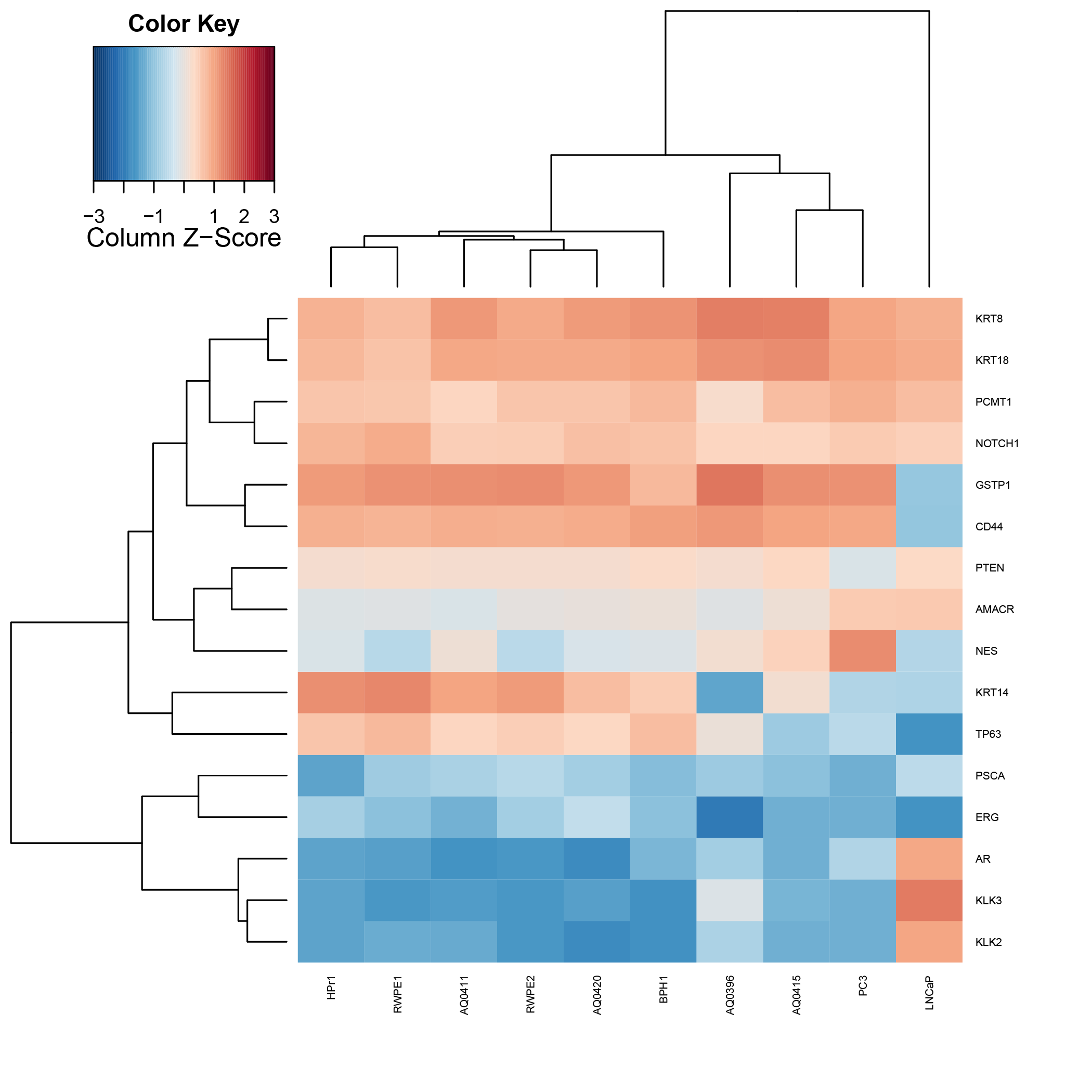

Supplement: Supplementary file 6 — Supplementary Figure 1 [file 41391_2023_679_MOESM6_ESM.png]

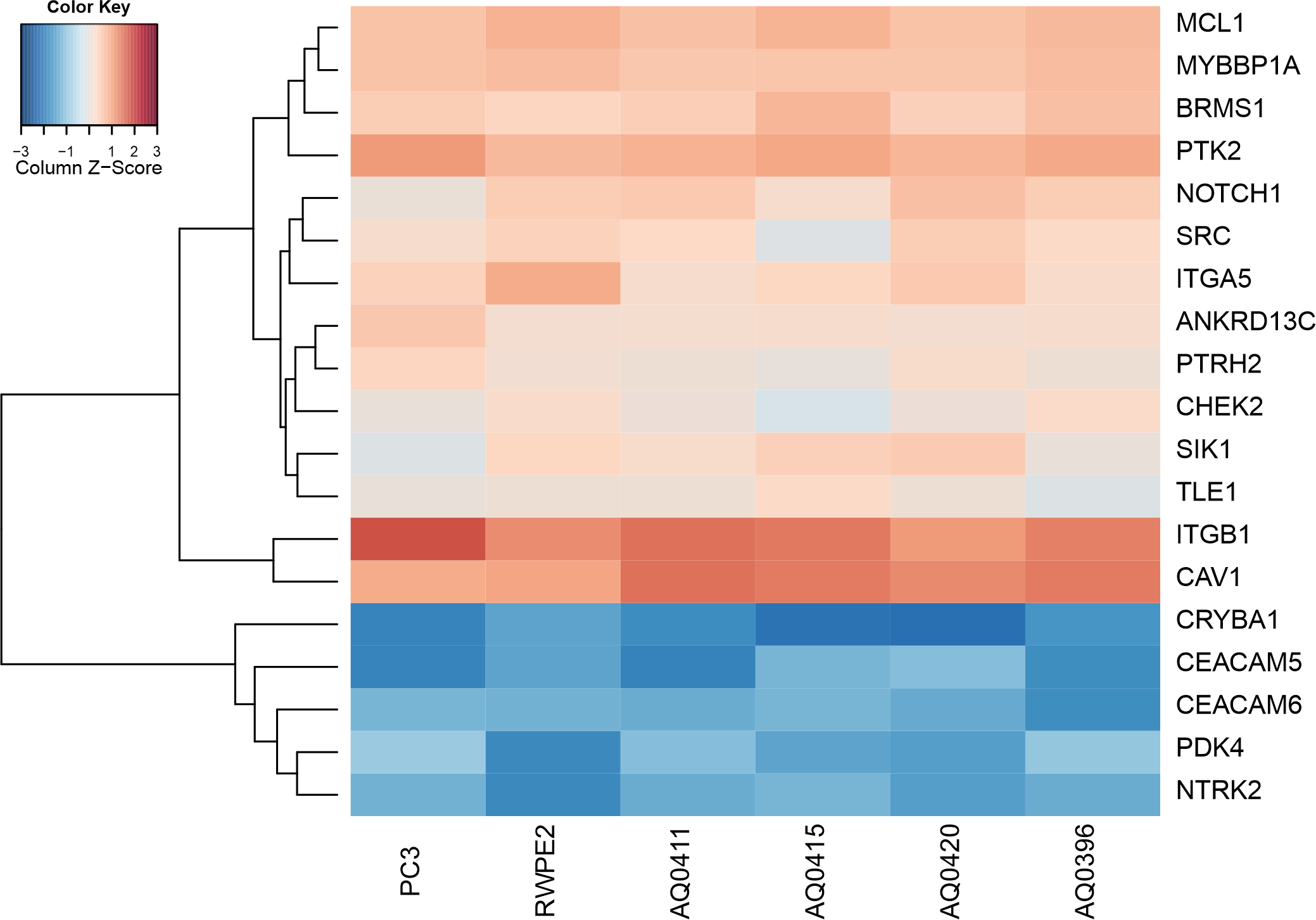

Supplement: Supplementary file 7 — Supplementary Figure 2 [file 41391_2023_679_MOESM7_ESM.png]
